# Insights into isotherms, kinetics, and thermodynamics of adsorption of acid blue 113 from an aqueous solution of nutraceutical industrial fennel seed spent

**DOI:** 10.1038/s41598-023-49471-w

**Published:** 2023-12-19

**Authors:** Syed Noeman Taqui, Akheel Ahmed Syed, Nabisab Mujawar Mubarak, Rizwan Abutaleeb Farade, M. A. Majeed Khan, Md. Abul Kalam, Mohammad Hadi Dehghani, Manzoore Elahi Mohammad Soudagar, Rauoof Ahmad Rather, Sathgatta Zaheeruddin Mohamed Shamshuddin, Rama Rao Karri

**Affiliations:** 1 Department of Chemistry, Bharathi College-Post Graduate and Research Centre, Bharathi Nagara, Karnataka 571422 India; 2 Centre for Advanced Research and Innovation, Glocal University, Delhi-Yamunotri Marg, SH - 57, Mirzapur Pole, Saharanpur District, Uttar Pradesh 247121 India; 3 Petroleum and Chemical Engineering, Faculty of Engineering, Universiti Teknologi Brunei, Bandar Seri Begawan, BE1410 Brunei Darussalam; 4 Department of Biosciences, Saveetha School of Engineering, Saveetha Institute of Medical and Technical Sciences, Chennai, India; 5 Department of Electrical and Electronics Engineering, Advanced Lightning, Power and Energy Research (ALPER), Faculty of Engineering, University Putra Malaysia, 43400 Serdang, Malaysia; 6 AIKTC, School of Engineering and Technology, Panvel, Navi Mumbai, India; 7 College of Science, King Saud University, Riyadh, 11451 Saudi Arabia; 8 School of Civil and Environmental Engineering, FEIT, University of Technology Sydney, Sydney, NSW 2007 Australia; 9 Department of Environmental Health Engineering, School of Public Health, Tehran University of Medical Sciences, Tehran, Iran; 10 Center for Solid Waste Research, Institute for Environmental Research, Tehran University of Medical Sciences, Tehran, Iran; 11 Institute of Sustainable Energy, Universiti Tenaga Nasional, Jalan IKRAM-UNITEN, 43000 Kajang, Selangor Malaysia; 12 Division of Environmental Sciences, Sher-E-Kashmir University of Agricultural Sciences and Technology, Srinagar, Jammu and Kashmir 190025 India; 13 Chemistry Research Laboratory, HMS Institute of Technology, Tumakuru, 572104 Karnataka India; 14 Faculty of Engineering, INTI International University, 71800 Nilai, Malaysia

**Keywords:** Environmental sciences, Environmental social sciences, Energy science and technology, Engineering, Materials science

## Abstract

Research studies have been carried out to accentuate Fennel Seed Spent, a by-product of the Nutraceutical Industry, as an inexpensive, recyclable and operational biosorbent for bioremediation of Acid Blue 113 (AB113) in simulated water-dye samples and textile industrial effluent (TIE). The physical process of adhesion of AB113 on the surface of the biosorbent depends on various parameters, such as the initial amount of the dye, amount and expanse of the biosorbent particles, pH of the solution and temperature of the medium. The data obtained was analyzed using three two-parameter and five three-parameter adsorption isotherm models to glean the adsorbent affinities and interaction mechanism of the adsorbate molecules and adsorbent surface. The adsorption feature study is conducted employing models of Weber-Morris, pseudo 1st and 2nd order, diffusion film model, Dumwald-Wagner and Avrami model. The study through 2nd order pseudo and Avrami models produced complementary results for the authentication of experimental data. The thermodynamic features, *ΔG*^*0*^, *ΔH*^0^, and *ΔS*^*0*^ of the adsorption process are acclaimed to be almost spontaneous, physical in nature and endothermic in their manifestation. Surface characterization was carried out using Scanner Electron Microscopy, and identification and determination of chemical species and molecular structure was performed using Infrared Spectroscopy (IR). Maximum adsorption evaluated using statistical optimization with different combinations of five independent variables to study the individual as well as combined effects by Fractional Factorial Experimental Design (FFED) was 236.18 mg g^−1^ under optimized conditions; pH of 2, adsorbent dosage of 0.500 g L^−1^, and an initial dye concentration of 209.47 mg L^−1^ for an adsorption time of 126.62 min with orbital shaking of 165 rpm at temperature 49.95 °C.

## Introduction

Polyamide fibres are frequently dyed with classified bisazo dye, Acid Blue 113 (AB113), to get a deep shade of navy blue on cotton, wool, and silk. Little information about its discoloration, degradation and adsorption from textile industrial effluent (TIE) has been reported. Various techniques, methods, and procedures have been reported in the literature, which are listed as electrocoagulation^1^, photocatalytic degradation^2,3^, biological–chemical procedures^4–13^, ultrasound methods with low frequency^14^, sono catalytic procedures^15,16^, and nanomaterials^17,18^. Various inorganic and nanomaterials are reported, of which activated carbon is widely used in textile industries^5,19,20^. These methods suffer from serious drawbacks such as the undesirable increase in operating expenses and overhead costs in the installation of plant and/or equipment, formation of degradation of the dye resulting in possible more toxic and hazardous secondary and tertiary pollutants, regeneration of the adsorbent resulting in high environmental and economic impact, inconsistency and large variation of the composition of the effluent, unwanted generation of the sludge are the few problems associated with the existing methods^21,22^.

Nutraceutical Industrial Spent (NIS) has been researched to be supplemented as composite filler material used in the construction sector^23,24^, the state-of-the-art investigations advocate the use of NIS in the form of an adsorbent serving in poisonous residual dye bioremediation^25–37^. In the recent past, the implementation of NIS to design and demonstrate circular economics in laboratory experiments was proposed in our research school^29^.

In the present scenario, a focused study has been conducted to establish the practicability of Nutraceutical Industrial Fennel Seed Spent (NIFSS) as a low-cost biosorbent for the remediation of AB113 in an aqueous fluid. The features affecting the process, adsorbent quantity and particle size, pH, dye percentage volume, and solution temperature have all been investigated. A comparison was made between the isotherm models with two parameters, such as Langmuir, Freundlich, and Jovanovic and three-parameter developed by Brouers-Sotolongo, Vieth-Sladek, Redlich-Peterson, Toth, and Radke-Prausnitz. The study included pseudo 1st and 2nd order, Dumwald-Wagner, Avrami model and Weber-Morris kinetic models of film diffusion. Investigations were conducted on thermodynamic parameters, including *ΔG*^*0*^, *ΔH*^*0*^, and *ΔS*^*0*^. The distinctive feature of this research is the use of NIS, an economical and environmentally beneficial biosorbent, to reduce dye toxicity caused by AB113 in wastewater from the textile sector. Finally, modelling studies and examining the cost of adsorbent renewal were conducted to complement the experimental results.

## Experimental

### Materials

Sigma-Aldrich, USA, provided AB113. Also referred to as Neutral Blue 5R. Its chemical formula is C_32_H_21_N_5_Na_2_O_6_S_2,_ and its molecular weight is 681.65. Its maximum absorbance (λ_max_) is 566 nm, as described in the Sigma-Aldrich catalogue.

### Material synthesis and characterisation

#### Synthesis

NIFSS is produced as a by-product after removing oleoresin from fennel seeds purchased from a nearby factory. The NIFSS was exposed to sunshine to dry, then was pulverized and processed in a ball mill, then sieved into the following particle sizes (X μm) following ASTM standards: X ≤ 90 μm; 90 μm ≤ X ≤ 125 μm; 125 μm ≤ X ≤ 177 μm; 177 μm ≤ X ≤ 355 μm; 355 μm ≤ X ≤ 500 μm and 500 μm ≤ X ≤ 710 μm. Several trials have been carried out for NIFSS ranging from 125 to 177 μm. The selected range is about ASTM standard, which specifies mesh 80-size particles.

#### Morphology interpretation

NIFSS surface study was conducted; LEO 435 VP model, Japanese make SEM was used. FTIR determined adsorbent functional groups; samples with and without CR adsorbed were examined through a spectrometer. The Inter-spec 2020, available by Spctro Lab, UK, was employed for FTIR analysis. To compute the NIFSS charge, the point of zero charge (PHZ) was determined.

#### Experimentation

The trials with different conditions were run in batches. The test batch constituted flasks of 250 ml, containing 50 ml of AB113 aqueous solution and 50 mg NIFSS. The AB113 was used in a concentration of 100 mg L^−1^. The flask contents were shaken for three hours at 165 rpm using a thermal control shaker. Parametric evaluation was conducted, including NIFSS dose (0.025, 0.050, 0.100, 0.150, 0.200, 0.300 g 50 mL^−1^), AB113 dye for range 25–150 ppm in increments of 25 ppm; higher concentration ranges 200–500 mg L^−1^ with 100 ppm increment and pH of solution was varied from 2 to 12. These metrics were investigated at three temperature conditions: 30 °C, 40 °C and 50 °C. The samples were centrifuged for five minutes to remove any particle matter. UV spectroscopy determined absorption features; a US-made Perkin Elmer-Lambda 25 spectrometer was employed. The UV spectrum at 566 nm was studied. The dye adsorption was monitored by mixing distilled water with adsorbent, including AB113, with no adsorbent. The dye quantity adsorbed was calculated at equilibrium using Eq. (1)

1 $${\mathfrak{q}}_{o}= \left({C}_{i}-{C}_{o}\right)\frac{\vartheta }{\omega }$$

where *ν* = volume (L), *ω* = adsorbent weight (g), and *C*_*i*_ and *C*_*o*_ are concentrations at the initial and equilibrium phase (mg L^−1^) of the AB113 dye, respectively.

The kinetic features were evaluated on similar lines, contrary to the part that samples were used at predetermined time duration and phase. The dye quantity utilized was predetermined; the amount of dye adsorbed instantly was computed using Eq. (2),

2 $${\mathcalligra{q}}_{t}= \left({C}_{i}-{C}_{t}\right)\frac{\vartheta }{\omega }$$

where dye quantity at instant t, *C*_*t*_ (mg L^−1^). The dye amount included initially for every litre content of the solution is 50 mg, 100 mg and 150 mg, with an adsorption duration of 60 min (5 min intervals). The adsorbent-adsorbate quantity ratio was obtained using NIFSS in 50 mL dye solution; the adsorbate quantity varied from 0.500 to 6.000 g L^−1^ to attain equilibrium. Further pH effect was examined; NIFSS, about 50 mg and dye, 50 mL in 200 mg L^−1^ concentration, were agitated employing a rotatory vibrator. The test procedure was repeated for solution pH 2–12 after 140 to 150 min of steady agitation at a speed of 165 rpm. A double-beam UV/Vis spectrophotometer was used to quantify the dye concentration at 566 nm. HCl and NaOH were suitably diluted to alter the pH. Using a pH meter, the solution's pH was determined, and the equation was used to determine the degree of dye loss (3).

3 $$\mathrm{Dye \, removal \, efficiency \% }= \frac{\left({{\text{C}}}_{{\text{i}}}-{{\text{C}}}_{{\text{o}}}\right)}{{{\text{C}}}_{{\text{i}}}}\times 100$$

The results of each biosorption experiment were carried out in the lab in triplicate, and they are shown as averages of the three trials.

#### Adsorption isotherms

The extensively used models for studying the adsorption equilibrium at ambient temperature have been incorporated; isotherm models include the Langmuir, Freundlich, and Jovanovic models. The others are Radke–Prausnitz, Brouers–Sotolongo, Redlich–Peterson, Toth and Vieth–Sladek models. This analysis provides a basic means to visualize the adsorption mechanism. Information on the adsorbent's affinities, surface characteristics, and interaction mechanisms can be gleaned from the findings of parameters examined in various models.

#### Adsorption kinetics

Kinetic models define the adsorption limit rate; experimental data is used to project this through pseudo 1st and 2nd-order equations providing the data fitting. The diffusion phenomenon was examined, and the effects were evaluated employing models like Weber–Morris, film diffusion and Dumwald–Wagner. The nonlinear least-squares method was used to fit each model. The experimental data fit obtained by appropriate model equations; formulate the parameter controlling the underlying mechanism.

#### Thermodynamic parameters

Understanding a process's energy and entropy can assist one in comprehending its viability and how the adsorption process works. The thermodynamic features specify the energy content and range of the process; the kinetic model, data and process rate information provide the thermodynamic parameter determination entropy and enthalpy change (*ΔS*^*0*^ and *ΔH*^*0*^), along with *ΔG*^*0*^, which is free energy change.

#### Parameter statistical analysis

Numerous parameters control the process of adsorption: process period ($\overline{A }$), heat range ($\overline{B }$), dye intake volume ($\overline{C }$), size of the adsorbent particle ($\overline{D }$), the volume of adsorbent ($\overline{E }$) and solution pH ($\overline{F }$). The capacity of adsorbent can be enhanced by optimizing these regulating parameters. The solution is shaken well at a speed stirring rate of 165 rpm; an orbital shaker is used. The experiment was conducted to record the extreme responses of the six distinguished parameters identified. The regression analysis of the obtained data is done, which is used for the graphical representation of the recorded data.

## Results and discussion

### Adsorbent morphological analysis

**Figure 1:**
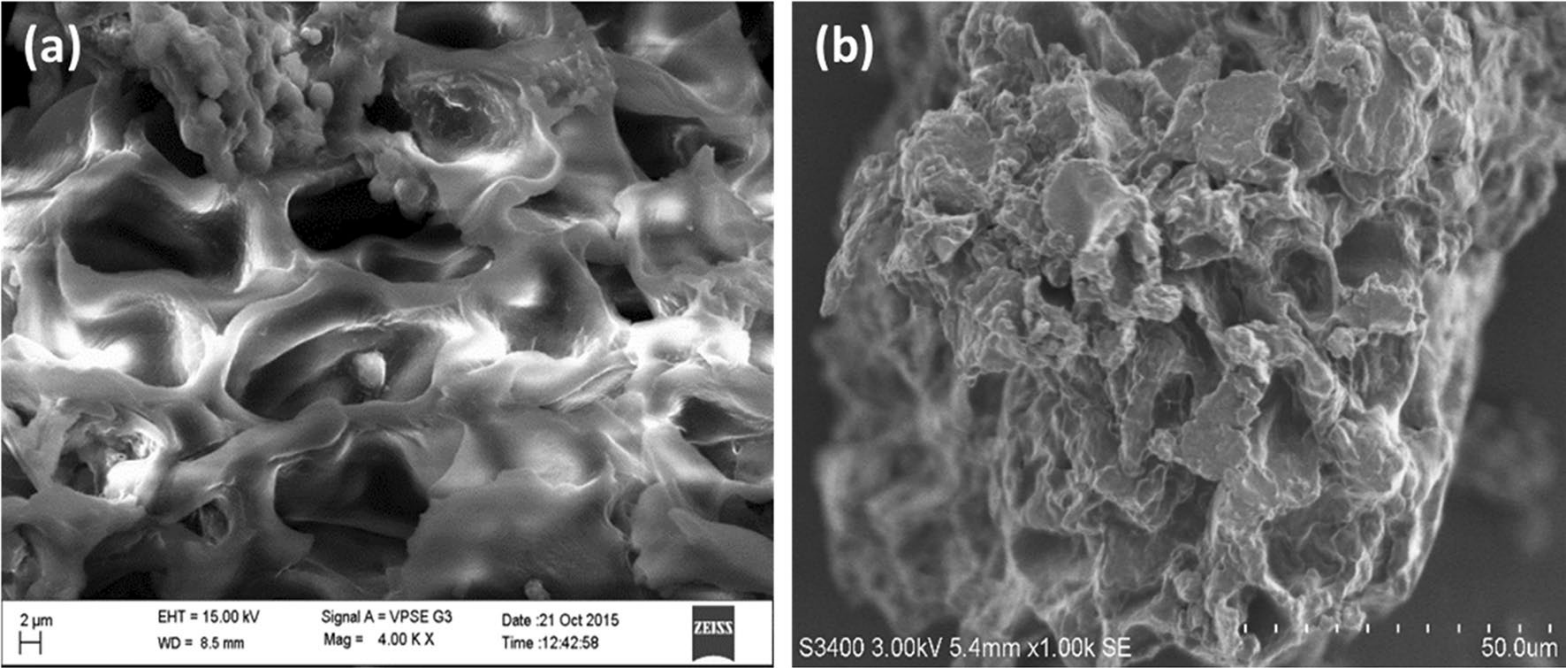
(**a**) NIFSS SEM image; (**b**) AB113-NIFSS SEM images.

Because of cellulose presence along with lignocellulose complexes, NIFSS has a fibrous and amorphous appearance. SEM analysis of the NIFSS surface revealed a convoluted porous structure (Fig. 1a). When the AB113 dye is absorbed, it is seen that some of the pores are filled, covering the particle with a thin layer (Fig. 1b).

**Figure 2:**
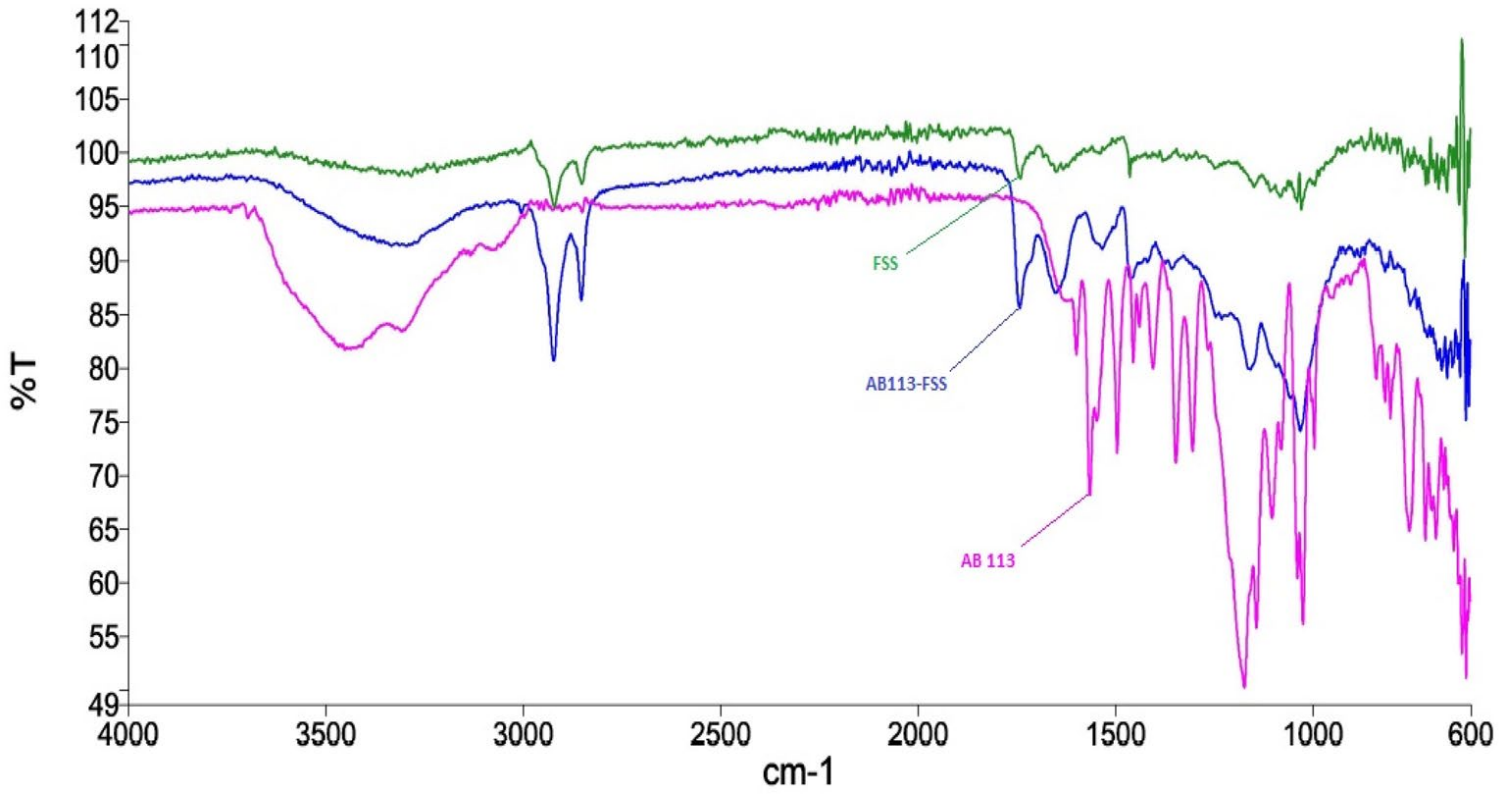
Adsorption FTIR spectrum analysis.

The NIFSS IR spectra reveals the present functional groups (Fig. 2). The IR spectrum, through 3100–3500 cm^−1^, shows cellulose presence (hydroxyl group) along with molecules of water adsorbed. A low band of 3000 cm^−1^ is identified for the C–H stretch; a 1600 cm^−1^ band is likely noticed for the C–O group's stretching. Bands at 1360, 1310, 1290, 1250 and 1020 cm^-1^ resemble C–O–C stretch. It was observed that bands created by N–H stretching caused by the presence of NH_2_ in the dye, ranging through 3200–3550 cm^−1^, and NIFSS hydroxyl group bandwidth decreased, projecting the development of hydrogen bonds. Furthermore, the dye adsorption on NIFSS is confirmed by peak elimination for N–N stretching in AB113 dye at 1500 cm^−1^. The point of zero charge was found to be pH 7.10.

### Dye intake volume and pH effect

**Figure 3:**
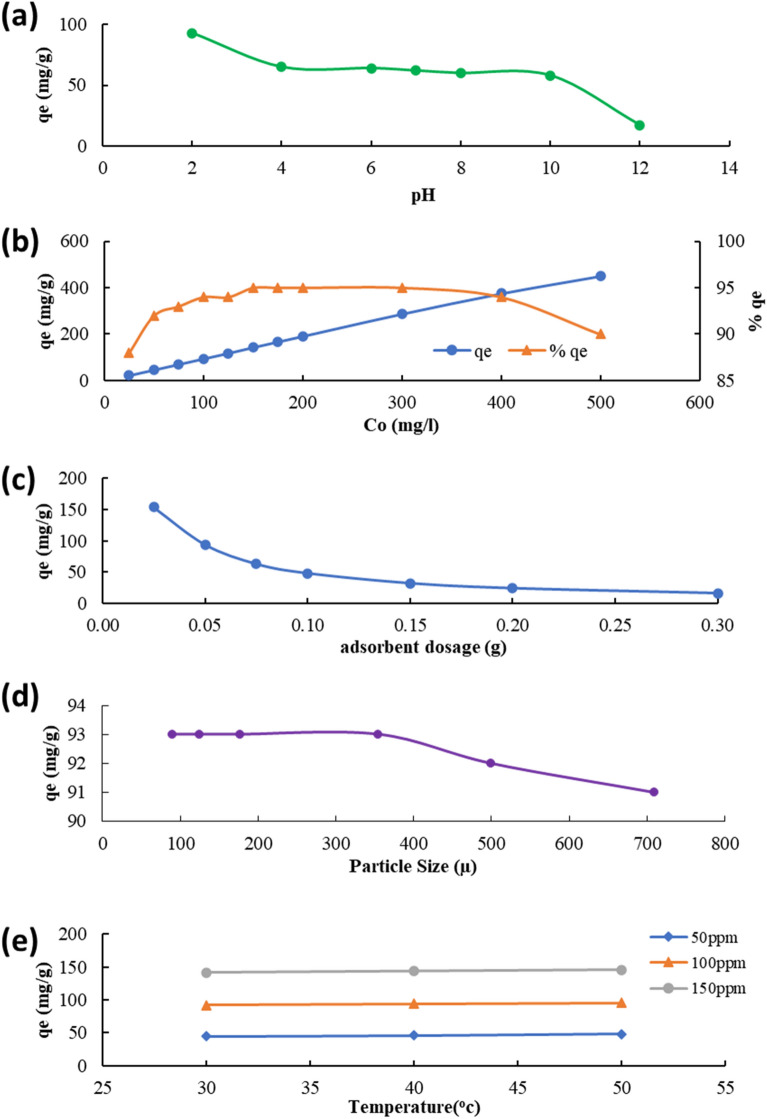
Parametric effect on adsorption at equilibrium (**a**) Solution pH, (**b**) Dye initial quantity (q_o_% considered), (**c**) Volume of adsorbent, (**d**) Size of particle and (**e**) temperature.

To achieve significant adsorption, the ideal parameters must be identified. The adsorption capacity is affected by the solution pH; it projects the properties possessed by the adsorbent surface and specifies the ionic content of the solution. The adsorption capacity was maximum when pH was 2 (Fig. 3a), and it significantly decreased between pH values of 2 and 8, again at pH 10 and even more at pH 12. It then further decreased beyond pH 12. The greatest amount of AB113 dye that NIFSS could remove was at solution pH 2 (*q*_*o*_ = 93.00 mg g^−1^), with the intake quantity being 100 mg L^−1^. A peak adsorption of 50 mg L^−1^ was achieved. The quantity of adsorption lowered as the intake volume increased. The variation is depicted in Fig. 3b.

### Effect of adsorbent dosage

The adsorption progress for an intake amount of adsorbate is driven by the volume of adsorbent, making it an important parameter to investigate. The dye absorption in 50 mL solution with 0.02–0.30 g adsorbent has been studied. The observations recorded reveal with adsorbent dosage increment, the amount of AB113 dye removal also increased. As the adsorbent dose was raised, more AB113 dye was adsorbed onto the NIFSS. The adsorption was not very significant beyond a limiting dose value. The bonding of dye molecules to the adsorbent surface was observed at the equilibrium phase. These details are presented in Fig. 3c.

### Particle size

The extent of adsorption was determined, considering neutral pH at a dye intake of 100 mg L^−1^. The particle sizes investigated are x ≤ 90 µm; 90 µm ≥ x ≤ 125 µm; 125 µm ≥ x ≤ 177 µm; 177 µm ≥ x ≤ 355 µm; 355 µm ≥ x ≤ 500 µm and 500 µm ≥ x ≤ 710 µm. An increment in particle size reduced the adsorbed quantity of dye (Fig. 3d). The observation is consistent with the anticipated outcomes because surface area always decreases as adsorbent particle size increases. As per the ASTM standards, a mesh size of 80 is preferred in composite manufacturing, so producing fine mesh is not economically viable and time-consuming; a sieve size of 125 µm ≥ x ≤ 177 µm is selected as ideal in the experiments.

### Effect of temperature

Another aspect that affects the adsorption process is temperature. Figure 3e displays the outcomes of the adsorption experiments, which were conducted at 30 °C to 50 °C with three different dye concentrations. It is apparent that the adsorption capacity gradually diminishes as the temperature rises, signifying the endothermic process. It has been reported^38^ that adsorption can be elevated at rising temperatures if dye molecules mobility is increased. This is attributed to decreased kinetic energy and paced diffusion of intra-particles.

### Isotherm analysis of adsorption

The features of adsorption isotherm are crucial for describing the interactions between adsorbate molecules and adsorbent surfaces. The isotherm model proposed by Langmuir perceives adsorption as taking place on a single layer; similar areas of energy uniformity are present on the adsorbent surface, presenting as adsorption sites. Langmuir equation^39^ is represented as follows:

4 $${\mathfrak{q}}_{o}=\frac{{Q}_{m}{K}_{a}{C}_{o}}{1+{K}_{a}{C}_{o}}=\frac{{Q}_{m}{K}_{a}{C}_{o}}{{R}_{L}}, where {R}_{L}=\frac{1}{1+{K}_{a}{C}_{o}}$$

*C*_*o*_ = dye equilibrium state in solution (mg L^−1^), and *q*_*o*_ = dye quantity adsorbed at equilibrium phase (mg g^−1^). *Q*_*m*_ = constant of Langmuir (L mg^−1^) and is the slope of *q*_*o*_ vs C_o_. K_a_ = ability to adsorb in a monolayer (mg g^−1^); it is determined through intercept of *q*_*o*_ vs C_o_. The equilibrium phase of AB113 dye was evaluated at bulk 25–500 mg L^−1^. The computations produce *Q*_*m*_ = 709.42 mg g^−1^, which is a value surpassing than expected for the current isotherm. Though *R*^*2*^ = 0.95, it deliberates proper data fit.

The separating element *R*_*L*_ is a crucial feature in Eq. (4)^40^. The adsorption status is favourable if *R*_*L*_ is less than unity and conversely unfavourable if it is greater than unity. The system adsorption is termed neutral when *R*_*L*_ = 1, and for *R*_*L*_ = 0, the process is irreversible. The computed range of *R*_*L*_, from 0.049 to a maximum of 0.204, resembles proper adsorption. It can be deduced from the drop in *R*_*L*_ value for concentration rise initially relates to the factual declaration of extensively efficient adsorption. The *Q*_*m*_ and *q*_*o*_ values calculated have more deviation; thus, different models have been analyzed to obtain alternative outcomes.

The isotherm model of Freundlich was incorporated^41^; the empirical formula considers heterogeneous adsorption surfaces. Equation (5) describes how the capacity of NIFSS to adsorb is related to the equilibrium dye concentration of AB113:

5 $${\mathfrak{q}}_{o}={K}_{f}{{C}_{o}}^{1/{n}_{f}}$$

where *K*_*f*_ and *n*_*f*_ are Freundlich constants. *K*_*f*_ = capacity of adsorption in mg/g and n_f_ = intensity of adsorption in (mg/L)^-1/n^. These values are obtained from a plot of ln (q_o_) vs. ln (C_o_). The intercept provides the *K*_*f*_ value, and the slope gives *n*_*f*_*.* The process quality classification is based on *n*_*f*_*;* adsorption is chemisorption if the intensity factor is less than unity and physisorption is greater than unity. For n_f_ equal to unity, the process is linear. The computed values *n*_*f*_ = 1.757 and *n*_*f*_^*−*1^ = 0.569 indicate the process being physisorption and support the Langmuir results. The data *R*^*2*^ = 0.89 does not provide a proper fit for this isotherm, complementing the linear nature of the process. The data derived from Langmuir and Frenudlich isotherms favour the adsorption of AB113 onto NIFSS through physical interactions and thus confirms that the process is physisorption. However, the above two isotherms investigated do not contribute to the demonstration of homogeneity or heterogeneity of the process. This has prompted the authors to incorporate models to achieve a better data fit.

**Figure 4:**
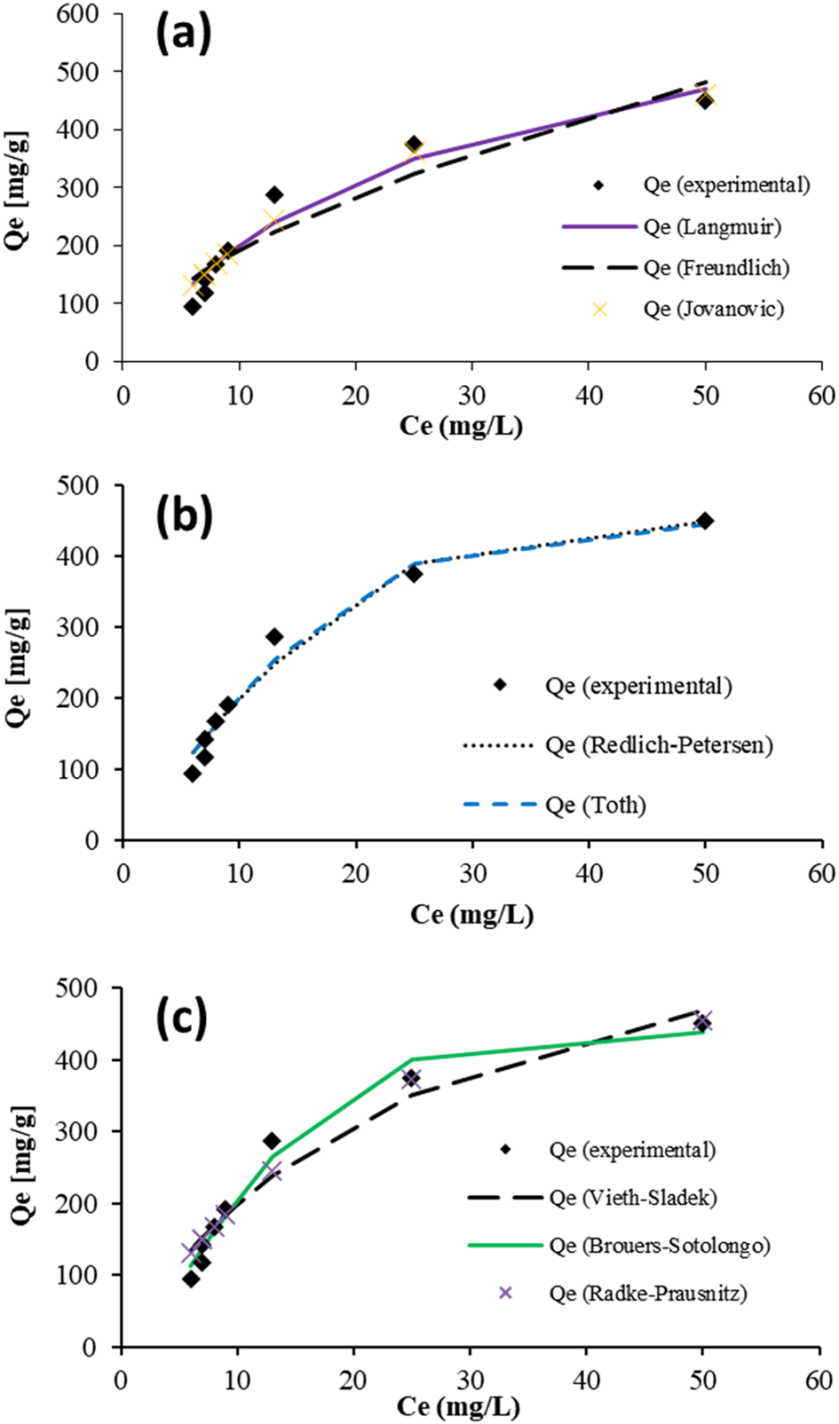
Several two- and three-parameter isotherms adsorption models.

The model proposed in Eq. (4) was improvised by Jovanovic^42^. It is expressed through Eq. (6),

6 $${\mathfrak{q}}_{o}={Q}_{m}\left[1-{e}^{{K}_{j}{C}_{o}}\right]$$

where *K*_*j*_ is termed the Jovanovic constant, the model depicts the formation of a single layer; no interactions laterally were observed. This model deviates from the Langmuir model because of *K*_*j*_*.* Dye adsorption at equilibrium was experimentally identified to be 93.00 mg g^−1^. This value relative to *Q*_*m*_ = 499.48 mg g^−1^ is considerably low. The data fit obtained is more compact, contrary to that of Langmuir isotherm. The variation is presented in Fig. 4.

The dye and adsorbent interaction (AB113 and NIFSS) features are described as linear, physical and beneficial. This characterization is being concluded concerning the two-parameter models discussed above (Tables 1 and 2). The authors have conducted an extensive study involving models based on three parameters. This study has been undertaken to imbibe a more in-depth understanding of the adsorption of dye onto the adsorbent. The further sessions are discussions on the various models and result interpretation.

**Table 1:** Calculated isotherm parameters for two parameters.

Langmuir		Freundlich		Jovanovic	
*Q*_*m*_	709.42	*K*_*f*_	52.04	*Q*_*m*_	499.48
*K*_*s*_	0.039	*n*_*f*_	1.757	*K*_*j*_	0.051

**Table 2:** Computation of three variables isotherm features.

Three parameter isotherms									
Redlich–Peterson		Toth		Radke–Prausnitz		Vieth–Sladek		Brouers–Sotolongo	
A_RP_	21.2	Q_m_	453.4	Q_m_	6,319,680	Q_m_	709.4	Q_m_	438.5
B_RP_	0.001	n_T0_	3.316	K_rp_	3.87E−06	K_VS_	1E−07	K_BS_	0.021
g	1.91	b_T0_	28,432.810	m_rp_	5118.073	β_VS_	0.039	α	1.469

The findings through Langmuir and Freundlich are elaborated and improvised by including a correction factor “g”. Redlich–Peterson proposed *g* = 1 concerning the Langmuir equation and g = 0 for the Freundlich isotherm; the governing equation is given as;

7 $${\mathfrak{q}}_{{\text{o}}}={A}_{rp}{C}_{o}/1+{B}_{rp}{C}_{o}^{g}$$

Redlich–Peterson constants^43^ are indicated as *A*_*rp*_, *B*_*rp*_ and *g.* The value of 1.910 was computed for “*g”*; this describes adsorption to tread through the Langmuir model.

The isotherm of Brouers–Sotolongo expresses the power and dissemination of active areas of adsorption. The parametric representation is by terms *K*_*bs*_ and *η*, respectively; these are referred to as Brouers–Sotolongo constants^44^. The equation formulated is given as follows.

8 $${\mathfrak{q}}_{o}={Q}_{m}\left\{\left[1-{e}^{-{K}_{bs}{C}_{o}^{\alpha }}\right]\right\}$$

*Q*_*m*_ = 438.50 mg g^−1^ is higher than 93.00 mg g^−1^ computed for this model. The *R*^*2*^ value is about 0.98, and the curve plotted conforms with the experimental results. This is presented in Fig. 4.

The isotherm proposed by Vieth–Sladek accounts for the dye molecules' adherence to the adsorbent surface^45^. The model is mathematically represented by Eq. (9),

9 $${\mathfrak{q}}_{o}={K}_{vs}{C}_{o}+\left(\frac{{Q}_{m}{\beta }_{vs}{C}_{o}}{1+{\beta }_{vs}{C}_{o}}\right)$$

where Vieth–Sladek constants are specified as *K*_*vs*_ and *β*_*vs*_*.* The values obtained experimentally project an optimum data fit for *R*^*2*^ = 0.95 *SSE* = 6247.2 and λ^2^ = 38.85. The *Q*_*m*_ value predicted according to this model is 709.4 mg g^−1^. This isotherm defines rates of solid diffusion through transient adsorption. The results reveal a strong resemblance to test data when the λ^2^ value is minimal. The test results are tabulated in Table 3 and graphically shown in Fig. 4.

**Table 3:** Model fitting statistical factors.

Isotherms	Langmuir	Freundlich	Jovanovic	Redlich–Peterson	Toth	Radke–Prausnitz	Vieth–Sladek	Brouers–Sotolongo
SSE	6247.2	11,894.9	4884.6	3281.4	2822.1	4087.7	6247.2	2092.8
χ^2^	38.852	64.909	33.026	21.349	18.944	28.689	38.852	11.292
*R*^*2*^	0.95	0.89	0.96	0.97	0.98	0.97	0.95	0.98

The process evaluation is further done using the Toth model, a scientifically approved methodology that extends the Langmuir model^46^. The Toth isotherm describes the systems possessing heterogeneous adsorption. For *R*^*2*^ = 0.98, the test data obtained is represented in Fig. 4. The maximum adsorption amount tabulated is about 6,319,680 mg g^−1^. This value is the same as obtained using the Radke–Praustnitz model^47^. These values are unusual, deducing that both models do not fit the experimental results.

In conclusion, higher-order equations are used in the models to explain the adsorption mechanism. *R*^*2*^ value alone cannot establish the validity of data fitting because it only applies to linear models. To obtain reliable results, *χ*^*2*^ values are considered that produce small values. The output will be adverse if there is a similarity between the model computed and experimental data. Table 3 presents values *Q*_*m*_, *R*^*2*^ and χ^2^ evaluated for all models considered. The variations in the values tabulated projecting the adsorption process are motivating to devise models to provide reliable outputs.

### Study of kinetic models

The present section deals with the kinetic aspect of adsorption. This study facilitates the parametric determination of adsorption control. The study was conducted for 50, 100 and 150 ppm dye volumes at 303 K, 313 K and 323 K process temperatures. The non-linear analysis of kinetic models^48–52^ was conducted. The controlling factors computed are tabulated in Table 4.

**Table 4:** Parameters for absorption kinetics models that were theoretically anticipated and experimentally determined.

Initial Concentration	Temp	*Qe*_*expt*_ (mg g^−1^)	Pseudo first order				Pseudo second order			
(ppm)	(K)		*Qe*_*pred*_ (mg g^−1^)	*k*_*1*_	R^2^	χ^2^	*Qe*_*pred*_ (mg g^−1^)	*k*_*2*_	R^2^	χ^2^
50	46	86	44.65	6.44E−01	0.38	0.10	45.22	6.95E−02	0.78	0.10
	47	89	45.82	7.97E−01	0.28	0.04	46.12	1.39E−01	0.66	0.02
	48	92	45.57	6.69E−01	0.29	0.18	46.10	8.13E−02	0.57	0.11
100	93	128	89.98	3.59E−01	0.78	0.59	93.37	9.59E−03	0.97	0.08
	95	130	92.38	9.24E+01	0.77	0.79	65.88	1.71E−03	0.76	1.65
	96	133	92.68	3.51E−01	0.75	0.78	96.43	8.69E−03	0.97	0.10
150	144	169	141.28	9.38E−01	0.20	0.04	141.80	8.09E−02	0.58	0.02
	145	172	142.27	9.42E−01	0.41	0.02	142.59	1.21E−01	0.55	0.01
	146	175	142.55	1.11E+00	0.03	0.06	142.84	1.50E−01	0.15	0.05

**Figure 5:**
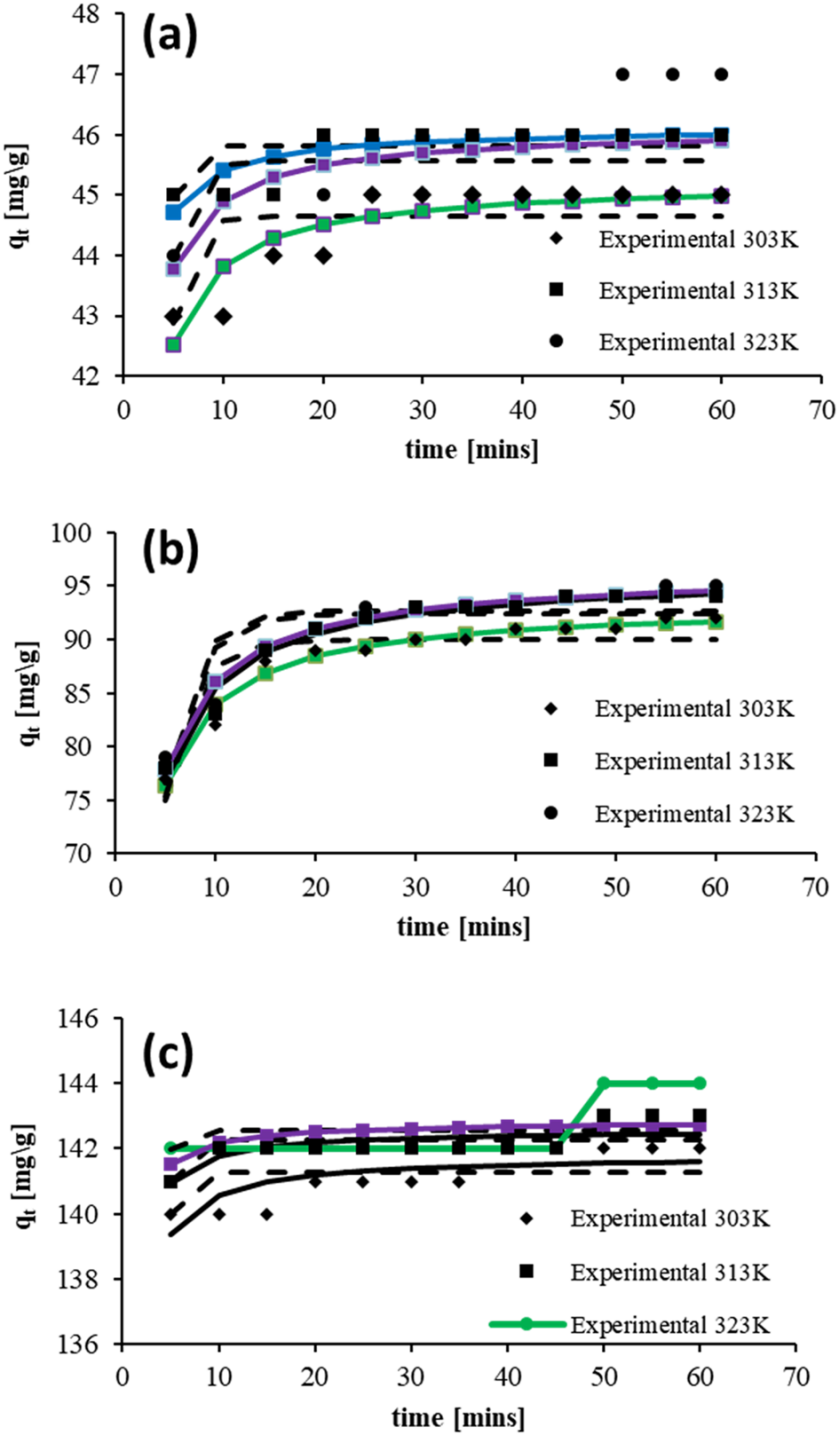
Initial AB113 dye concentrations of (**a**) 50, (**b**) 100, and (**c**) 150 ppm of the kinetic model on the NIFSS system at various temperatures.

The above data observation of determination coefficient (*R*^*2*^) and chi-square (χ^2^) reveals compatibility among the second-order pseudo model and experimental data at all dye concentrations, as shown in Fig. 5. The process of adsorption was noticed to slow down after maximum attainment. The process was enhanced with temperature rise. This specifies the no-limit rate nature of adsorption, which occurs in levels, starting with the diffusion of solute molecules onto the NIFSS solid surface through the pores.

**Figure 6:**
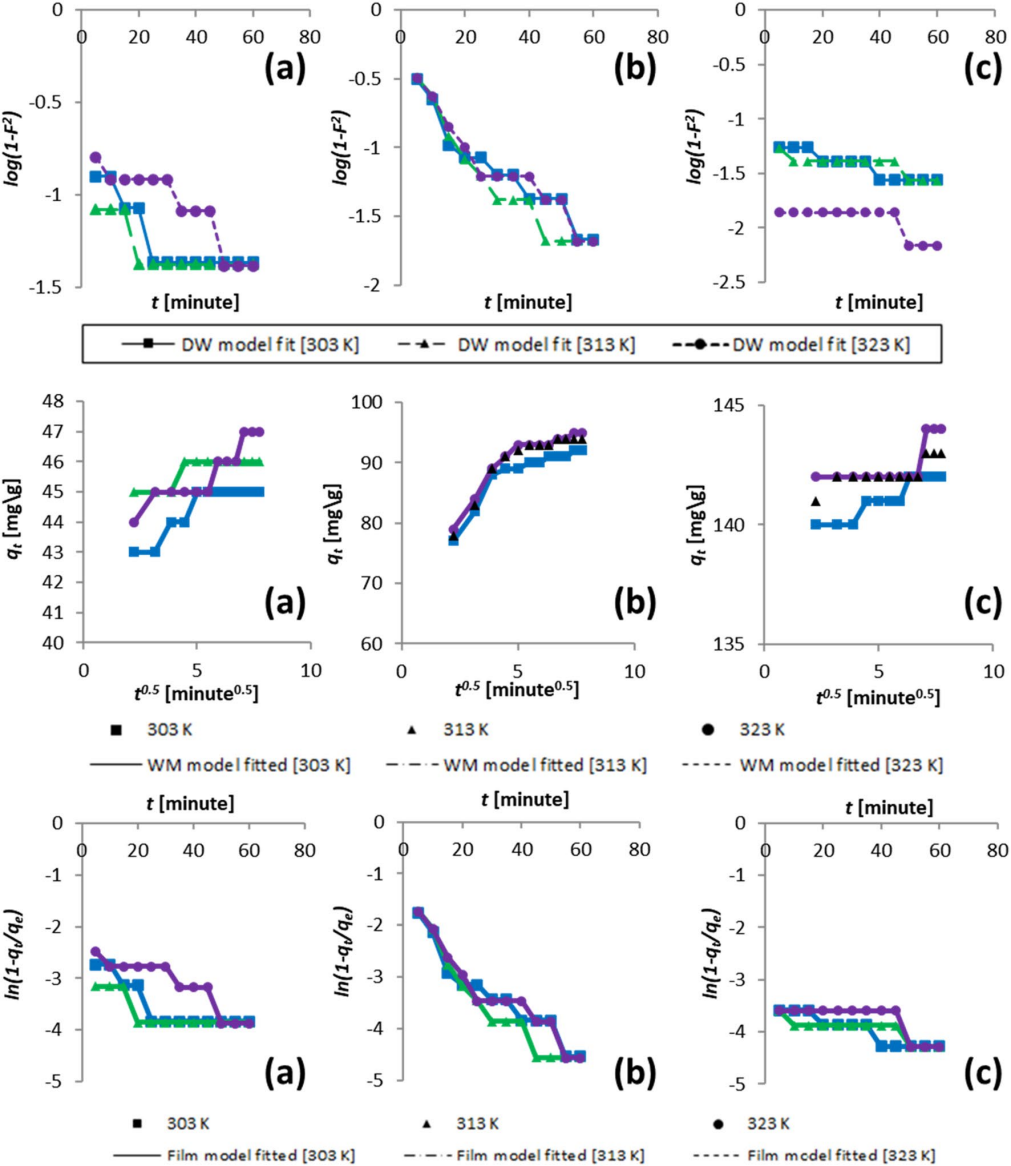
Kinetic model initial concentration data fitting for (**a**) 50 ppm, (**b**) 100 ppm, and (**c**) 150 ppm.

The diffusion features tabulated in Table 5 are altered by the Dumwald–Wagner model, which evaluates adsorption's actual rate constant (K). This is depicted in Fig. 6. The period of contact variation for solute uptake has been noticed to be *t*^*1/2*^*.* Weber-Morris reported this; a graph is shown in Fig. 6. The curve obtained is a linear straight line through the starting point. The curve slope defines the rate constant. Numerous mechanisms govern the adsorption process.

**Table 5:** Models of diffusion parametric calculation.

Initial volume	Temp	Film diffusion model		Weber–Morris model		Dumwald–Wagner	
(ppm)	(K)	*R*^*|*^ (min^−1^)	*R*^*2*^	*k*_*ist*_ (mg/g s^0.5^)	*R*^*2*^	*K* (min^−1^)	*R*^*2*^
50	303	0.0212	0.69	0.40	0.78	0.002	0.69
	313	0.0131	0.57	0.21	0.66	0.035	0.77
	323	0.0255	0.86	0.52	0.86	0.008	0.43
100	303	0.0448	0.92	2.29	0.79	0.044	0.92
	313	0.0519	0.91	2.59	0.79	0.051	0.91
	323	0.0468	0.92	2.54	0.82	0.046	0.92
150	303	0.0154	0.88	0.45	0.89	0.015	0.88
	313	0.0099	0.69	0.27	0.67	0.808	0.69
	323	0.0131	0.57	0.35	0.46	0.013	0.57

The results obtained experimentally dictate linear trends at different stages and concentrations of the solute. It is summarized that process rates were high at minimal concentrations of 50 ppm and reduced temperatures. The process progresses linearly till equilibrium is attained. The process rate at elevated temperatures is found to be more linear. The adsorption rate varies less obviously at higher solute concentrations (150 ppm). This is especially true when Boyd et al.'s method fits higher temperature data. The excerpts from Fig. 6 indicate proper data fit at *R*^*2*^ and χ^2^ values; the constant of fluid film diffusion *R*^*I*^ is obtained. These proceedings reveal that the adsorption rate is slowed due to diffusion when the temperature is higher. The discussion above dictates that process rate hampering is caused by diffusion. The solute forms a coating on the surface of the particles right away after it absorbs quickly, which explains why the absorption rates shift over time.

### Adsorption thermodynamics

**Figure 7:**
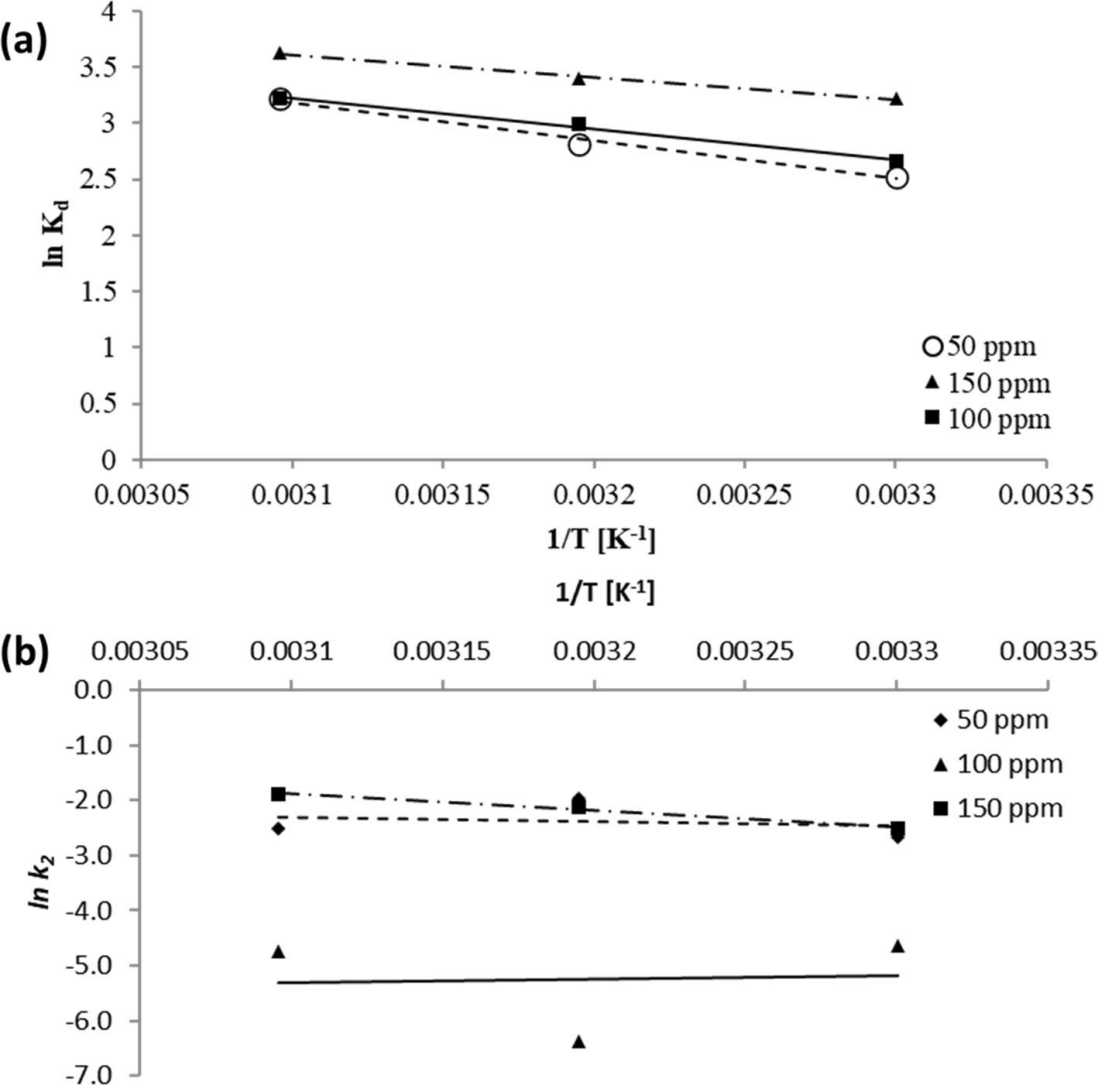
(**a**) Thermodynamic constant at equilibrium v/s 1/T, (**b**) The kinetic constant of pseudo-second order v/s 1/T.

Energy and entropy determination are critical parameters to evaluate the process occurrence. Energy change indicated by *G°* adjudicates the process volatility, which signifies considerable process activity when *∆G°* is negative. These features are computed employing equations of Van't Hoff and Gibbs–Helmholtz.

10 $${K}_{L}=\frac{{C}_{ac}}{{C}_{o}}$$

11 $$\Delta G^\circ =-RT{\text{ln}}{K}_{L}$$

12 $${\text{ln}}{K}_{L}=\Delta S^\circ /R-\Delta H^\circ /RT$$

where *K*_*L*_ = thermodynamic constant of equilibrium. C_ac_ = concentration of dye initially in the solution. Figure 7a and b illustrate calculating H° using ln(*K*_*L*_) and *1/T* graph slope. Using the intercept, S° is determined. Similarly, the slope and intercept of ln(K_L_) and *1/T* graph are used to find E_a_. These plots are Van’t Hoff graphs.

Table 6 provides the estimations for the thermodynamic parameter. The positive enthalpy change describes the endothermic feature of adsorption, while negative *G°* dictates the process's spontaneous nature. The adsorption process is extensive at temperatures considered, depicted from negative *∆G°.* It can be deduced that at higher temperatures, adsorption increases, as *∆G°* is observed to reduce with temperature rise. An increased surface activity is noticed for *∆S°* (positive) and strong affinity of the AB113 dyes towards the adsorbent.

**Table 6:** AB113-NIFSS thermodynamic features.

Initial volume	Temperature	*ΔG°*	*ΔS°*	*ΔH°*	*ln A*	*E*_*a*_
(ppm)	(K)	(kJ mol^−1^)	(J^−1^ mol^−1^ K)	(kJ mol^−1^)		(kJ mol^−1^)
50	303	− 6.36	113.68	28.14	0.27	6.91
	313	− 7.32				
	323	− 8.64				
100	303	− 6.69	97.51	22.81	− 7.35	− 5.46
	313	− 7.79				
	323	− 8.64				
150	303	− 8.11	81.05	16.47	7.51	25.19
	313	− 8.86				
	323	− 9.73				

Since enthalpy changes for chemical reactions are typically > 200 kJ mol^−1^, lower enthalpy change specifies the physical nature of the adsorption process. At all concentrations considered, the activation energy is approximated within -5.46 to 25.19 kJ mol^−1^. This range is computed using the Arrhenius equation. The second-order kinetic model constant is applied for the range determination (Table 6).

### Optimization

**Figure 8:**
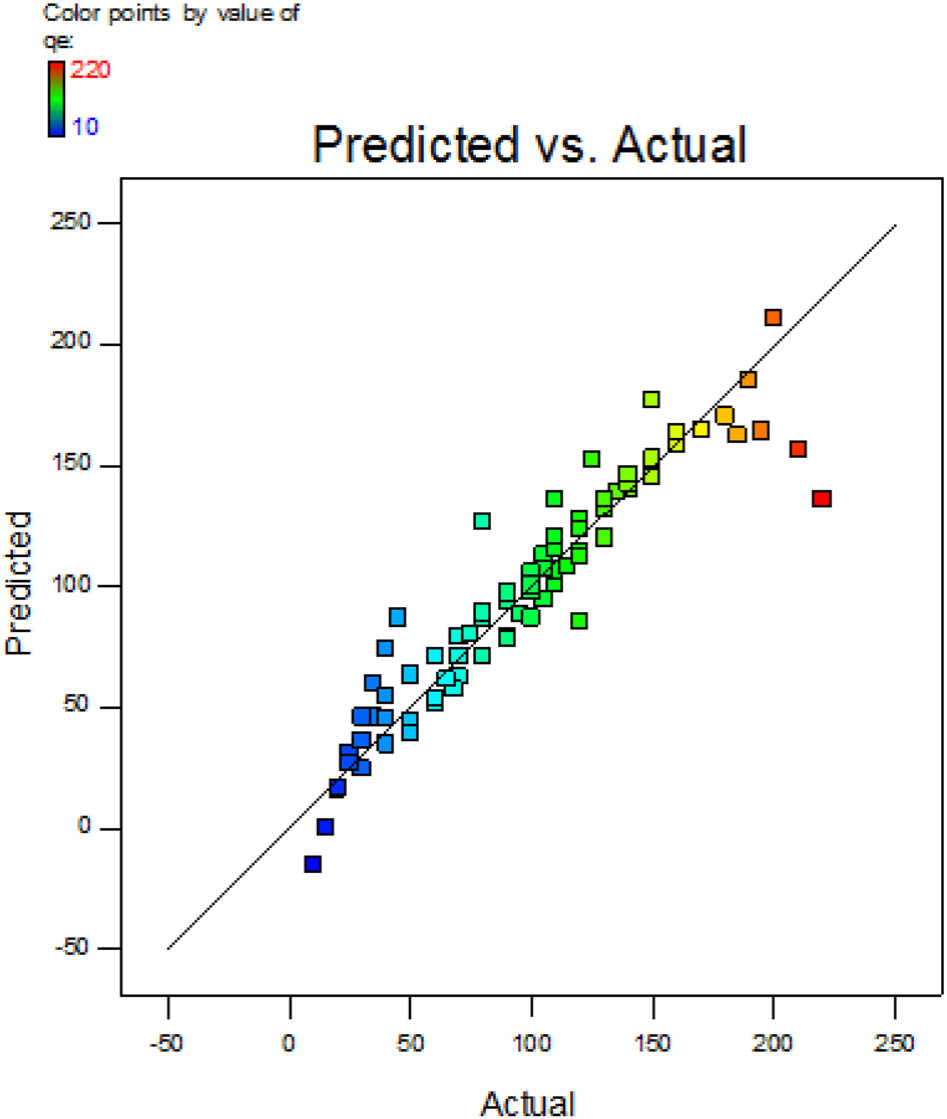
Data comparative plot.

The regression model of fraction factorial is incorporated for obtaining experimental data and fitting it. This was achieved in two stages. The actual versus anticipated values comparison graph (Fig. 8) correlates experiment results and estimated values. The process regression study is computed using Eq. (13)

13 $${\text{Adsorption }} = { 89}.{4 } + \, \left( {\Omega _{{1}} } \right) \, + \, \left( {\Omega _{{2}} } \right) \, + \, \left( {\Omega _{{3}} } \right) \, {-}{ 59}.{5 }{-} \, \left( {\Omega _{{4}} } \right) \, {-} \, \left( {\Omega _{{5}} } \right) \, {-} \, \left( {\Omega _{{6}} } \right) \, + \, \left( {\Omega _{{7}} } \right) \, {-} \, \left( {\Omega _{{8}} } \right) \, + \, \left( {\Omega _{{9}} } \right) \, {-} \, \left( {\Omega _{{{1}0}} } \right)$$

where, Ω_1_ = 61.56.6 × Ḁ, Ω_2_ = 28 × Ḅ, Ω_3_ = 113.3 × Ḉ, Ω_4_ = 21.2 × Ḛ, Ω_5_ = 8.4 × ḀḄ, Ω_6_ = 63.11 × Ḁ^2^, Ω_7_ = 0.004 × Ḅ^2^, Ω_8_ = 20.55 × Ḉ^2^, Ω_9_ = 52.87 × Ḍ^2^, Ω_10_ = 48.37 × Ḛ^2^.

By employing interaction products from multiple regression analysis to maximize the second-order polynomial equation, the optimal values of the variables may be found following FFED. The optimum parameters considered are pH 2, the volume of adsorbent = 0.500 g L^−1^, an adsorption period of 126.62 min, and dye quantity initially = 209.47 mg L^−1^ agitated at 165 rpm in an orbital shaker. The process temperature is 49.95 °C. An adsorption of 236.18 mg g^−1^ is achieved.

Five independent factors were used in various experiment combinations to explore the individual and combined effects. The quadratic regression analysis's results for Analysis of Variance (Table 7) show how important each factor's individual and combined effects are. The results obtained exhibited a confidence rate of 95%; the p-value was lower than 0.05%. The factors investigated, namely Ḁ, Ḅ, Ḉ, Ḍ, ḀḄ, Ḁ^2^ and Ḉ^2^, were observed to be important, while Ḛ has been termed mildly significant; all other parameters were considered negligible. The statistical model appears meaningful according to an adjusted *R*^*2*^ value of 85.8%. The model effectively fits the data obtained through experimentation with a coefficient of variance of 19.50% and a high *R*^*2*^ value. There is a discernible rise in adsorption capacity with increasing process time and initial dye concentration values. However, when their total impact is considered, it is almost nonexistent.

**Table 7:** ANOVA for fractional factorial experimental design.

Parameters	Σ^2^	D.O.F	Σ^2^_mean_	*F* value	*P-*value
Model	179,497.3	11	16,317.93	44.4735	< 0.0001**
Ḁ	47,015.75	1	47,015.75	128.1385	< 0.0001**
Ḅ	16,988.58	1	16,988.58	46.30131	< 0.0001**
Ḉ	56,236.35	1	56,236.35	153.2687	< 0.0001**
Ḍ	13,717.61	1	13,717.61	37.38649	< 0.0001**
Ḛ	2829.371	1	2829.371	7.711274	0.007**
ḀḄ	113.685	1	113.685	0.310	0.580
Ḁ^2^	21,545.17	1	21,545.17	58.72001	< 0.0001**
Ḅ^2^	0.000159	1	0.000159	4.33E−07	0.999477
Ḉ^2^	760.4977	1	760.4977	2.072689	0.154545
Ḍ^2^	3042.676	1	3042.676	8.292622	0.005**
Ḛ^2^	4037.602	1	4037.602	11.00423	0.001**
Residual	24,950.13	68	366.9136		
Lack of fit	17,675.13	65	271.925	0.112134	0.99995
Total	7275	3	2425		

^+^Suggestive significance (*P* value: 0.05 < *P* < 0.10).

*Moderately significant (*P* value: 0.01 < *P* ≤ 0.05).

**Strongly significant (*P* value: *P* ≤ 0.01).

Consequently, the text does not contain any contour plots or 3D graphics. As shown in the equation, regression coefficient values show how the parameter affects adsorption capacity. Positive numbers signify a progressive effect; a longer adsorption time produces a much higher adsorption capacity^53^.

**Figure 9:**
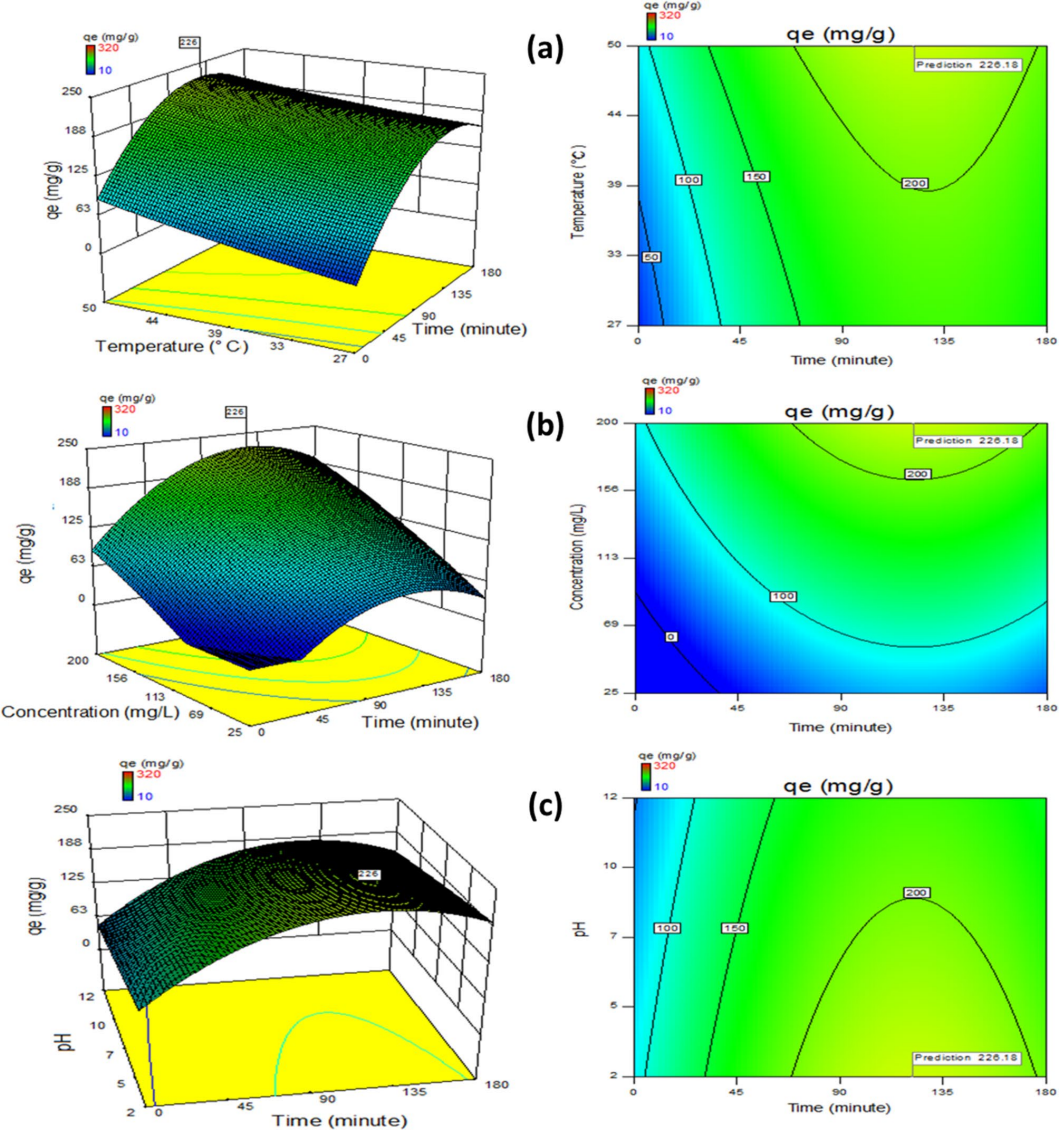
Adsorption capacity fluctuation, time versus plots (**a**) temperature, (**b**) concentration and (**c**) pH.

Additionally, the interaction effects between the two factors were described through 3D surface response and contour graphs, plotted for variables with other features kept constant. In addition to allowing for calculating the ideal condition, statistical process optimization also identifies how the process conditions affect adsorption. The synthesis of 3D plots representing variations of the adsorption period under numerous controllable depicts a favourable impact. Increasing the absorption capacity by lengthening the experiment's duration, particle size, and dye concentration is feasible. At an absorption duration of 125.62 min, the maximum absorption is reached. The peak adsorption capacity prediction and different parametric interaction influences on the adsorption process were computed by incorporating a quadratic model drafted to enhance the process, which revealed good results (Fig. 9a–c).

The various interpretations discussed idealize NIFSS as an adsorbent for AB113 dye extraction. Adsorption appears to be a primarily physical process that is thermodynamically possible. Additionally, by comprehending the dynamics and employing FFED, we have optimized the process from the standpoint of the variables that have a big impact on it, opening the door for process scaling.

### Method application for TIE

Wastewater produced by the various processes used in the textile industry has a variety of components, including high concentrations of suspended particulates, an extremely variable pH, a variable temperature, strong colour, and COD concentration^54^. As a result, the matrix effect makes it challenging to locate a particular dye in industrial waste^55^. A straightforward approach for AB113 dye remediation from industry effluents like textile and water comparison was established.

#### Textile industrial effluent (TIE)

The textile plant in the neighbourhood, having two work shifts, was identified for a sample collection of effluents. Six random TIE samples were taken at the pipe's end, 10-L plastic containers. Three samples were taken consecutively during the first and second shifts throughout three working days. To achieve consistent concentration, all samples of textile manufacturing effluent were shifted to a 100-L barrel and physically swirled. The final product served as the control TIE sample for the analyses. Standard procedures were used for sampling, preparing, and preserving the effluent samples collected from the industries^56^.

#### AB113 dye synthesis using distilled H_2_O

A 2-L flask was filled with two grams of the AB113 dye. To achieve a constant concentration, the dye was continuously stirred into distilled water (Solution 1).

#### AB113 dye synthesis in effluent from the textile industry

AB113 dye weighing 2 g was put into a 2-L flask. TIE was used to dissolve the dye and to adjust the solution to the proper consistency. The resulting mixture was swirled to achieve a consistent concentration (Solution 2).

#### Blank experiment

The process involves magnetic stirring using a Teflon 5 cm coat magnet. The solution comprises 5 g NIFSS in distilled H_2_O of about 500 ml, taken in a conical flask of 1 L volume. The stirring was carried out for 15 min and later filtered through filter paper (Whatman No. 42). The collected filtrate matches the TIE dye and the corrected residue.

#### Procedures

##### Absorbance validation

The TIE fractional amount is filtered using a Buchner funnel device and No. 42 Whatman filter paper. The filtrate's absorbance was measured using a UV–Vis spectrophotometer at three absorbances. A suitable dilution's absorbance was measured first to obtain a concentrated solution's absorbance. Subsequently, the product value of absorbance and dilution factor is determined. Furthermore, this process was repeated in the absorbance measurement of solutions 1 and 2.

##### AB113 dye molar absorption coefficient (ε) computation

**Figure 10:**
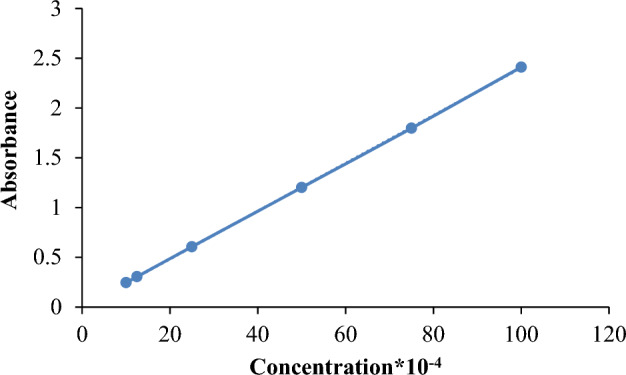
AB113 dye coefficient of molecular extinction determination.

AB113 dye was prepared in distilled water in dosages of 1.00 × 10^–4^; 1.25 × 10^–4^, 2.50 × 10^–4^, 5.00 × 10^–4^, 7.50 × 10^–4^, and 10.00 × 10^–4^, and at 566 nm absorbance was tested and recorded, taking distilled water as base reference (Fig. 10). The relationship between absorbance and concentration was graphed and calculated using the equation = A/cl, where for concentration “c”, the specific absorption “A” over 1-cm length can be determined from the graph slope. The unit quantities of concentration and absorbance over one unit length are also known as the absorbency index or specific absorption coefficient. In the latter scenario, the mean of six values for the AB113 dye was determined as follows:

$$\varepsilon_{AB113} = \, \varepsilon 1 \, + \, \varepsilon 2 + \, \varepsilon 3 + \, \varepsilon 4 + \, \varepsilon 5 \, + \varepsilon 6 \, / \, 6$$

$$\varepsilon_{AB113} = \, 2450 \, + \, 2440 \, + \, 2424 \, + \, 2402 \, + \, 2396 \, + 2411 \, /6 \, = \, 2421$$

**Figure 11:**
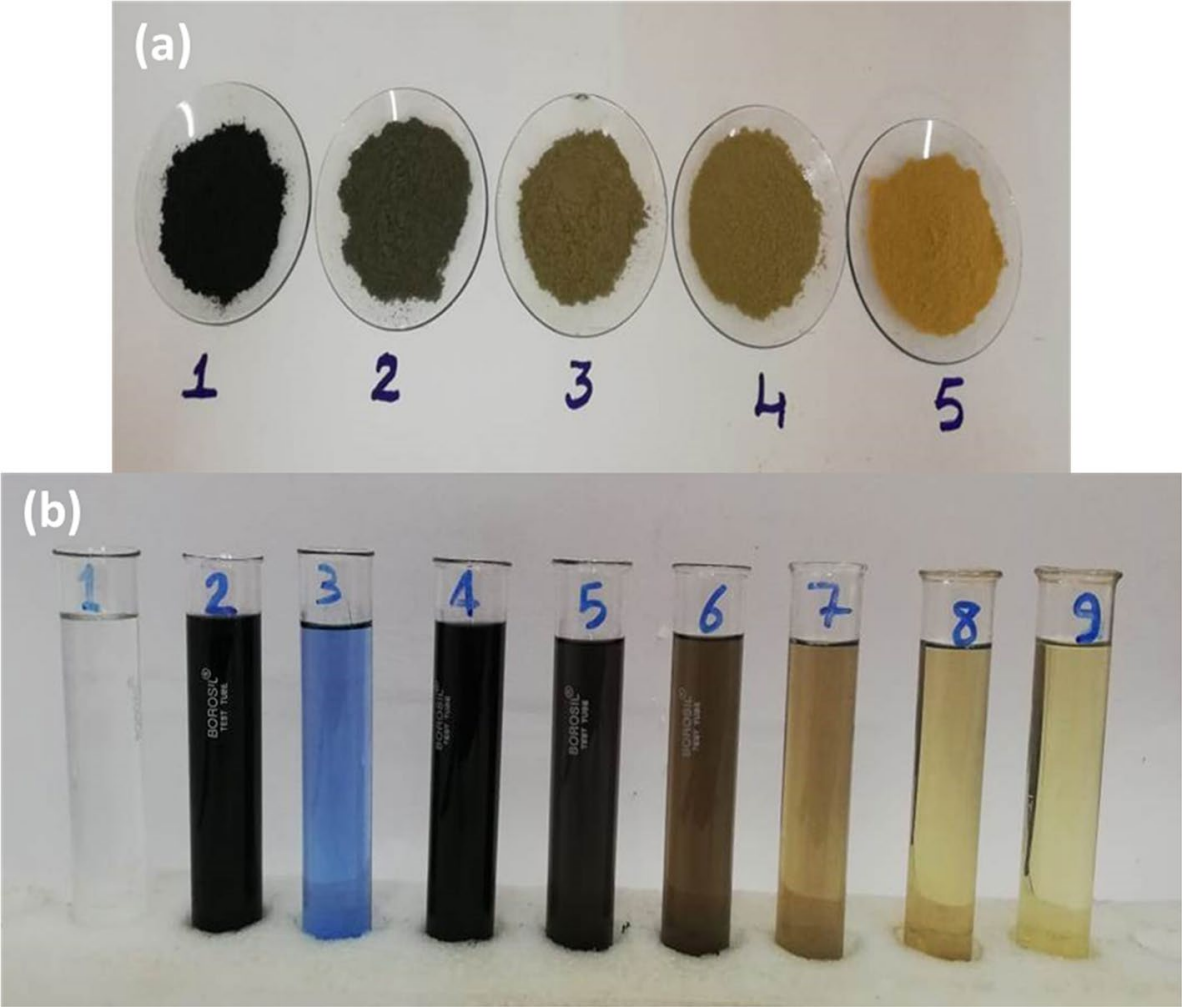
(**a**) Sample of filtered, dried NIFSS and AB113 mixture at 15-min intervals through 1–4. NIFSS sample in **5**; (**b**) Solution contrasts on adsorption prior and later: **1**. Distilled water; **2**. AB113 dye in distilled water; **3**. TIE; **4**. AB113 dye in TIE; **5**. Filtrate after dye adsorption on NIFSS after 15 min; **6**. 30 min; **7**. 45 min; **8**. 60 min; **9**. Filtrate of NIFSS in distilled water.

The ingredients in solution 2 adsorption process to NIFSS are shown in Fig. 11a and b. According to a preliminary investigation, the results desired can be improved by an increase in the quantity of basic components; adsorbent and adsorbate can be double scaled, while solution volume can be scaled to one. An intriguing finding was that Solution 2's absorbance decreased by roughly 32% compared to Solution 1 in this experiment. This might result from the dye being absorbed by many unidentified components found in TIE. Additionally, additional fresh adsorbent samples were found to improve the effectiveness of dye removal from TIE after every 15 min. After 15, 30, 45, and 60 min, the dye and associated compounds were recovered from Solution 2 to a degree of 63%, 77%, 89%, and 95% respectively. This discovery is consistent with the kinetic data, which show that the solute immediately develops a surface layer of particle film, thereby delaying diffusion and changing the absorption rates.

The experiment was scaled up to use a HDPE beaker's volume of solution 2 and NIFSS quantity as (1 L: 10 g, 2 L: 20 g and 5 L: 50 g). The procedures were performed with forcefully churned solutions with a magnetic stirrer, and the outcomes were nearly uniform. The mean of three outcomes from each experiment, performed in triplicate, is presented. The coefficient of variance of the results obtained was within ± 2%.

In short, the trial to experiment with an increased scale of three compared to the actual setup has yielded good results. The residue of industrial waste could contain a wide range of variables that make it difficult to evaluate the preliminary results precisely. This is a significant flaw in all such studies. The results may be narrowly focused by using a much bigger pilot research. Although scale limits have been highlighted, experimentation at increasing scales yields trustworthy process data, which encourages using the strategy for large-scale industrial development.

Production activities in textile industries lead to water pollution by transforming green and blue water into grey water footprints, resulting in freshwater depletion. Evaluation of water pollution is a precondition for sustainable water management, which is best achieved by water treatment processes. This paper presents a systemic approach by using myriad tons of spent generated in the Nutraceutical Industries to eliminate the toxic dyes from the effluents of textile industries. Resultant waste (dye-adsorbed nutraceutical industrial spent) is used as filler material to manufacture partially green composites using the waste from plastic industries^31,32,57^. The scheme fits into the concept of circular economy^29^, which aims for a plausible solution to address the issues of Sustainable Development Goals.

### Adsorbent regeneration and cost analysis

Reusing dye-loaded NIFSS and recovering the adsorbed material by regeneration is possible. This might not be cost-effective because the process and solvent costs will be more than the adsorbent used in the method (about $1 US for 10 kg NIFSS). Due to the enormous amount of environmental contaminants, it will also raise the E-factor, which is undesirable.

## Conclusion

NIFSS has been used as a biosorbent to detoxify toxic dyes from TIE to add value to the waste generated in the nutraceutical industries. AB113 can be efficiently eliminated from aqueous solutions using NIFSS with an absorption capacity of 236.18 mg g^–1^. The adsorption process of adsorbate onto absorbent is endothermic and spontaneous due to low enthalpy values. The SEM and FTIR spectra endorse that AB113 had been adsorbed onto NIFSS. The models studied confirm that the interaction between the adsorbate and the adsorbent is physical in nature and the reaction is endothermic. The results obtained matched the pseudo-second-order kinetic model. Utilizing spent from the nutraceutical industry as an adsorbent to reduce pollution is now possible in a cleaner, less expensive, and more effective method.

In conclusion, the rise and rise of the Nutraceutical Industries as a significant sector of the world economy has a parallel problem of generating myriad tons of spent and waste with no fertilizer, fuel or feed value. This has led to a serious environmental pollution problem. The never-ending list of innumerable dyes used in wide-ranging industries has resulted in an unprecedented demand for removing toxic and hazardous dyes and their degraded products from water bodies. The widely used activated charcoal as an effective adsorbent has serious limitations of high cost, which has inspired us to find a cheap alternative available in large quantities and fits into the concept of Circular Economy.

## Data Availability

The datasets used and analyzed during the current study are available from the corresponding author upon reasonable request.
