# Author Correction: Insights into isotherms, kinetics, and thermodynamics of adsorption of acid blue 113 from an aqueous solution of nutraceutical industrial fennel seed spent

**DOI:** 10.1038/s41598-024-53033-z

**Published:** 2024-01-30

**Authors:** Syed Noeman Taqui, Akheel Ahmed Syed, Nabisab Mujawar Mubarak, Rizwan Abutaleeb Farade, M. A. Majeed Khan, Md. Abul Kalam, Mohammad Hadi Dehghani, Manzoore Elahi Mohammad Soudagar, Rauoof Ahmad Rather, Sathgatta Zaheeruddin Mohamed Shamshuddin, Rama Rao Karri

**Affiliations:** 1 Department of Chemistry, Bharathi College-Post Graduate and Research Centre, Bharathi Nagara, Karnataka 571422 India; 2 Centre for Advanced Research and Innovation, Glocal University, Delhi-Yamunotri Marg, SH - 57, Mirzapur Pole, Saharanpur District, Uttar Pradesh 247121 India; 3 Petroleum and Chemical Engineering, Faculty of Engineering, Universiti Teknologi Brunei, Bandar Seri Begawan, BE1410 Brunei Darussalam; 4 Department of Biosciences, Saveetha School of Engineering, Saveetha Institute of Medical and Technical Sciences, Chennai, India; 5 Department of Electrical and Electronics Engineering, Advanced Lightning, Power and Energy Research (ALPER), Faculty of Engineering, University Putra Malaysia, 43400 Serdang, Malaysia; 6 AIKTC, School of Engineering and Technology, Panvel, Navi Mumbai, India; 7 College of Science, King Saud University, Riyadh, 11451 Saudi Arabia; 8 School of Civil and Environmental Engineering, FEIT, University of Technology Sydney, Sydney, NSW 2007 Australia; 9 Department of Environmental Health Engineering, School of Public Health, Tehran University of Medical Sciences, Tehran, Iran; 10 Center for Solid Waste Research, Institute for Environmental Research, Tehran University of Medical Sciences, Tehran, Iran; 11 Institute of Sustainable Energy, Universiti Tenaga Nasional, Jalan IKRAM-UNITEN, 43000 Kajang, Selangor Malaysia; 12 Division of Environmental Sciences, Sher-E-Kashmir University of Agricultural Sciences and Technology, Srinagar, Jammu and Kashmir 190025 India; 13 Chemistry Research Laboratory, HMS Institute of Technology, Tumakuru, 572104 Karnataka India; 14 Faculty of Engineering, INTI International University, 71800 Nilai, Malaysia

Correction to: *Scientific Reports* 10.1038/s41598-023-49471-w, published online 19 December 2023

The original version of this Article omitted an affiliation for Mohammad Hadi Dehghani. The correct affiliations are listed below.

Department of Environmental Health Engineering, School of Public Health, Tehran University of Medical Science, Tehran, Iran.

Center for Solid Waste Research, Institute for Environmental Research, Tehran University of Medical Sciences, Tehran, Iran.

In addition, Manzoore Elahi Mohammad Soudagar was incorrectly affiliated with ‘Center for Solid Waste Research, Institute for Environmental Research, Tehran University of Medical Sciences, Tehran, Iran’.

The original Article has been corrected.

